# 真性红细胞增多症诊断与治疗中国指南（2022年版）

**DOI:** 10.3760/cma.j.issn.0253-2727.2022.07.002

**Published:** 2022-07

**Authors:** 中华医学会血液学分会白血病淋巴瘤学组

自《真性红细胞增多症诊断与治疗中国专家共识（2016版）》[1]发布后，对真性红细胞增多症（polycythemiavera, PV）的诊断、预后分层、新药和传统治疗药物再评估的认识又有了长足的进步[2]–[3]。为给我国血液科医生提供规范化的临床实践指导，中华医学会血液学分会白血病淋巴瘤学组组织国内相关专家制定了本指南。

一、诊断程序

1. 病史采集：必须仔细询问患者年龄，有无血栓病史，有无心血管高危因素（如高血压、高血脂、糖尿病、吸烟和充血性心力衰竭），有无疲劳、早饱感、腹部不适、皮肤瘙痒和骨痛，以及活动力、注意力、此前1年内体重下降情况，有无不能解释的发热或重度盗汗及其持续时间，家族有无类似患者，有无长期高原生活史，有无呼吸系统和心血管系统疾病导致的慢性缺氧病史。患者初诊时及治疗过程中评估患者疗效时采用骨髓增殖性肿瘤总症状评估量表（MPN-SAF TSS，也称MPN-10）[4]对患者进行症状负荷评估。

2. 实验室检查：以下实验室检查应作为疑诊PV患者的必检项目[5]–[8]：①外周血细胞计数；②骨髓穿刺涂片和外周血涂片分类计数；③骨髓活检活组织切片病理细胞学分析，铁染色和网状纤维（嗜银）染色；④血清促红细胞生成素（EPO）、血清铁、血清铁蛋白水平、乳酸脱氢酶（LDH）、尿酸和肝功能测定；⑤JAK2 V617F和JAK2 exon 12基因突变检测（骨髓或外周血）；⑥肝脏、脾脏超声或CT检查。

对于血小板计数增高和（或）脾脏肿大或有临床不能解释的出血患者，建议进行凝血酶原时间（PT）、活化部分凝血活酶时间（APTT）、血管性血友病因子抗原结合试验（VWF∶CBA）和瑞斯托霉素辅因子活性测定。有家族病史者建议筛查EPOR、VHL、EGLN1/PHD2、EPAS1/HIF2α、HGBB、HGBA和BPGM等基因突变。二代测序（NGS）不作常规推荐，但采用包括LNK、CBL、TET2、ASXL1、IDH、IKZF1、EZH2、DNMT3A、TP53、NFE2、SF3B1、SRSF2和U2AF1等基因的套餐（panel）靶向测序结果有助于患者总生存（OS）、无纤维化生存和白血病转化率的评估，可酌情选择。

二、诊断标准

1. PV诊断标准：建议采用WHO（2016）标准[9]（表1）。

**表1:** WHO（2016）真性红细胞增多症诊断标准[9]

诊断需满足3项主要标准或前2项主要标准加1项次要标准
主要标准
① HGB>165 g/L（男性），>160 g/L（女性）或红细胞压积（HCT）>49％（男性），>48％（女性）或红细胞容量（RCM）升高
② 骨髓活检示与年龄不符的细胞过多伴三系增生（全骨髓增生），包括显著红系、粒系、巨核系增生并伴有多形性成熟巨核细胞（细胞大小不等）
③ 有JAK2V617F或JAK2第12号外显子基因突变
次要标准
血清促红细胞生成素（EPO）低于正常水平

2. 真性红细胞增多症后骨髓纤维化（post-PV MF）诊断标准：采用骨髓纤维化研究和治疗国际工作组（IWG-MRT）标准[10]。

主要标准（2条均需满足）：①此前按WHO诊断标准确诊为PV；②骨髓活检示纤维组织分级为2/3级（按0～3级标准）或3/4级（按0～4级标准）。

次要标准（至少符合其中2条）：①贫血或不需持续静脉放血（在没有采用降细胞治疗情况下）或降细胞治疗来控制红细胞增多；②外周血出现幼稚粒细胞、幼稚红细胞；③进行性脾脏肿大（此前有脾脏肿大者超过左肋缘下5 cm或新出现可触及的脾脏肿大）；④以下3项体质性症状中至少出现1项：过去6个月内体重下降>10％，盗汗，不能解释的发热（>37.5 °C）。

三、预后判断标准

PV患者确诊后，为了更好地指导治疗选择，应对患者的预后分组作出判断。

1. 血栓风险分组[5]–[8]：按年龄和血栓病史分为高危组和低危组：①高危组：年龄≥65岁和（或）此前有PV相关动脉或静脉血栓；②低危组：年龄<65岁和（或）此前无PV相关动脉或静脉血栓。

2. 生存预后分组：如果患者未行NGS，采用IWG-PV预后分组积分系统[11]，依据年龄（≥67岁，5分；57～66岁，2分）、WBC>15×10^9^/L（1分）和静脉血栓（1分），分为低危组（0分）、中危组（1或2分）和高危组（≥3分），中位OS时间分别为28、19、11年。如果患者行NGS，也可采用加入基因突变的预后分组积分系统，依据SRSF2基因突变（3分）、年龄>67岁（2分）、WBC≥15×10^9^/L（1分）、血栓史（1分），将患者分为低危组（0～1分）、中危组（2～3分）和高危组（≥4分），中位OS时间分别为24.0、13.1、3.2年。

3. post-PV生存预后分组：采用PV和ET继发骨髓纤维化预后模型（MYSEC-PM）[12]，依据确诊时年龄（分值为0.15×年龄）、HGB<110 g/L（2分）、外周血原始细胞比例≥3％（2分）、无CALR Ⅰ型突变（2分）、PLT<150×10^9^/L（1分）和有体质性症状（1分），将患者分为低危（<11分）、中危1（≥11分）、中危2（14分～<16分）和高危（≥16分）。

四、治疗

1. 治疗目标：PV的治疗目标是避免初发或复发的血栓形成、控制疾病相关症状、预防post-PV MF和（或）急性白血病转化。现阶段治疗策略主要依据患者血栓风险预后分组来加以制定。多血症期治疗目标是控制红细胞压积（HCT）<45％[13]。

2. 一线治疗选择：

（1）共存疾患和对症处理：有高血压、高血脂、糖尿病等共存疾患的患者应同时与相关科室配合积极进行相应处理，控制病情。皮肤瘙痒采用静脉放血/骨髓抑制药物常无效，由于热水洗澡可使之加重，因此，可告诫患者减少洗澡次数或避免用过热的水洗澡，阿司匹林和赛庚啶亦有一定疗效，但抗组胺药物无效。

（2）血栓预防：由于血栓是PV患者死亡的主要原因，因此，确诊患者均应进行血栓预防。首选口服低剂量阿司匹林（70～100 mg/d），不能耐受阿司匹林的患者可选用口服氯吡格雷75 mg/d或双嘧达莫25～50 mg每日3次[14]。

（3）静脉放血：一般来说，静脉放血开始阶段为每次300～450 ml，每周1次或2次，HCT降至正常（<45％）后可延长放血间隔时间，以维持红细胞数正常的状态。HCT>64％的患者初期放血间隔期应更短，体重低于50 kg的患者每次放血量应减少，对于有心血管疾患的患者放血应采用少量多次的原则。静脉放血可使头痛等症状得到改善，但不能降低血小板和白细胞水平，对皮肤瘙痒和痛风等症状亦无效。年龄低于50岁且无血栓病史患者可首选此种治疗方法。红细胞单采术可在短时间内快速降低HCT，必要时可以采用此治疗。反复静脉放血治疗可出现铁缺乏的相关症状和体征，但一般不进行补铁治疗。

（4）降细胞治疗：血栓预后分组为高危患者应予降细胞治疗[5]–[7]。血栓预后分组为低危组患者，出现对静脉放血不能耐受（反复出现放血后晕厥、有血液恐惧症或静脉通路非常困难）或需频繁放血、有症状或进行性的脾脏肿大（在除外post-PV MF前提下，在过去的1年内脾脏增大>5 cm）和持续性（3个月）白细胞计数>20×10^9^/L亦应采用降细胞治疗，在出现白细胞进行性（基数<10×10^9^/L时至少上升100％，或在基数>10×10^9^/L时至少上升50％）以及持续性（3个月）增高、PLT>1 500×10^9^/L和（或）发生与PV相关的出血时亦应考虑降细胞治疗，有严重的疾病相关症状（MPN10总积分≥20分或瘙痒≥ 5分）应推荐加入降细胞治疗的临床试验[7]。

羟基脲或常规剂型干扰素α（IFN-α）和长效INF-α（聚乙二醇干扰素α和聚乙二醇脯氨酸干扰素α）为任何年龄需降细胞治疗PV患者的一线药物[15]–[17]。年轻患者（<60岁）推荐首选干扰素。年长患者（>70岁）可考虑口服白消安（2～4 mg/d）[18]。

羟基脲起始剂量为30 mg·kg^−1^·d^−1^，口服，1周后改为5～20 mg·kg^−1^·d^−1^，需维持给药并调整用药剂量，联合静脉放血治疗（必要时采用红细胞单采术）可降低血栓并发症。

常规剂型IFN-α剂量为（9～25）×10^6^ U/周（分3次皮下注射）。用药6～12个月后，70％患者的HCT可获控制，20％的患者获部分缓解，10％无效；随着用药时间的延长，部分患者JAK2V617F负荷逐渐下降，达部分或完全分子学缓解；此外，还可使血小板计数、皮肤瘙痒和脾肿大得到显著改善[16]。聚乙二醇干扰素α起始剂量为45 µg每周1次，聚乙二醇脯氨酸干扰素α起始剂量为100 µg每2周1次。临床试验结果表明接受较长时间治疗（≥24个月）后长效干扰素可获得较好的症状改善、较高的血液学和分子学缓解率。相比于常规剂型IFN-α，长效干扰素不仅给药间隔时间延长，而且药物不良反应发生率和严重程度显著降低[14]–[15]。IFN-α的主要非血液学药物不良反应有甲状腺功能减低、抑郁等精神症状和自身免疫性疾病，拟接受干扰素治疗的患者应进行相关检查，以除外亚临床甲状腺功能异常、自身免疫和精神性疾患。

3. 二线治疗选择：约25％的患者在用羟基脲治疗期间可出现耐药或不耐受（表2）[19]，20％～30％的患者有干扰素治疗不耐受，这些患者可采用二线治疗。

**表2:** 真性红细胞增多症羟基脲治疗耐药或不耐受的判断标准[19]

①至少2 g/d羟基脲治疗3个月后，仍需放血以维持红细胞压积（HCT）<45％
②至少2 g/d 羟基脲治疗3个月后，仍不能控制骨髓增殖（PLT>400×10^9^/L及WBC>10×10^9^/L）
③至少2 g/d 羟基脲治疗3个月后，触诊的巨大脾脏未能缩小50％以上或脾大相关的临床症状未能完全缓解
④在使疾病达到完全或部分临床血液学反应所需的羟基脲最小剂量下，中性粒细胞计数（ANC）<1×10^9^/L或PLT<100×10^9^/L或HGB<100 g/L
⑤任何剂量羟基脲治疗下，出现小腿溃疡或其他不能接受的羟基脲相关非血液学毒性（皮肤黏膜表现、胃肠道症状、肺炎、发热等）

（1）芦可替尼：在一项国际、随机、开放标签、多中心Ⅲ期临床试验[20]中，依赖静脉放血治疗伴有脾脏肿大的PV患者随机接受芦可替尼（110例，起始剂量20 mg/d）或标准治疗（112例，医师根据情况选用羟基脲、干扰素、阿那格雷、来那度胺、沙利度胺或不予任何治疗），32周时芦可替尼组、标准治疗组的HCT控制率（HCT<45％）分别为60％、20％，脾脏容积减少35％的患者比例分别为38％、1％，完全血液学缓解率分别为24％、9％，症状下降50％的患者比例分别为49％、5％。据此结果，2014年12月芦可替尼被FDA批准用于治疗羟基脲疗效不佳或不能耐受的PV患者。PV患者治疗推荐起始剂量为20 mg/d。在开始治疗的前4周不要进行剂量调整，每次剂量调整间隔时间不应少于2周，最大剂量不超过50 mg/d。

芦可替尼最常见的血液学不良反应为3/4级的贫血、血小板减少以及中性粒细胞减少，但极少导致治疗中断[20]–[22]。治疗过程中血PLT<50×10^9^/L或中性粒细胞绝对计数（ANC）<0.5×10^9^/L、HGB<80 g/L应停药，出现贫血的患者可加用EPO或达那唑。停药应在7～10 d内逐渐减停，应避免突然停药，停药过程中推荐加用泼尼松20～30 mg/d。

（2）干扰素：羟基脲治疗期间出现耐药或不耐受患者可换用干扰素，如聚乙二醇干扰素α或聚乙二醇脯氨酸干扰素α。羟基脲耐药或不耐受患者换药是选用干扰素还是芦可替尼目前尚无共识，二者均可酌情选用。

（3）^32^P：静脉给予一次性^32^P 2～4 mCi常可使疾病得到很好的控制，间隔6～8周后可依首剂疗效再次给予。^32^P治疗最大的不良反应是远期发生治疗相关性白血病或骨髓增生异常综合征（MDS）及肿瘤。适合老年（>70岁）患者选用。

（4）白消安：2～4 mg/d，口服，几周后HCT下降的同时白细胞计数常降至正常，停药后血细胞计数维持正常几个月至几年不等。由于本药可致严重骨髓抑制，因此，用量不宜超过4 mg/d。

4. post-PV MF和白血病变患者的治疗：post-PV MF的治疗按原发性骨髓纤维化治疗原则，具体参考《原发性骨髓纤维化诊断和治疗中国指南（2019版）》[23]。白血病变患者按相应指南[24]原则处理。

5. PV患者妊娠期处理：计划妊娠的患者，至少3个月前停用所有可能致畸的药物。妊娠期间应由血液科医师和妇产科医师共同制定管理计划，用干扰素或静脉放血严格控制HCT<45％[25]–[26]。低危组患者妊娠期服用小剂量阿司匹林，分娩前2周至分娩后6周可改用低分子肝素，高危组患者妊娠期和分娩后6周采用小剂量阿司匹林和低分子肝素联合预防血栓。阿司匹林会小量分泌入乳汁，低分子肝素不经乳汁分泌，二者均不影响哺乳。羟基脲、干扰素等均会分泌入乳汁，因此用药期间禁止哺乳。

五、疗效判断标准

根据欧洲白血病网和骨髓增殖性肿瘤研究和治疗国际工作组2013年修订的PV疗效评价标准[27]（表3），主要包括临床血液学及骨髓组织学评价两方面。分子生物学疗效对于评价完全缓解（CR）或部分缓解（PR）不是必须的。完全分子生物学缓解（CRm）定义为：原先存在的异常完全消失。部分分子生物学缓解仅用于基线的等位基因突变负荷≥20％且等位基因突变负荷下降≥50％的患者。

**表3:** 真性红细胞增多症的疗效标准[27]

疗效	标准
完全缓解（CR）	以下4条必须全部符合：
	①包括可触及的肝脾肿大等疾病相关体征持续（≥12周）消失，症状显著改善（MPN-SAF TSS积分下降≥10分）；
	②外周血细胞计数持续（≥12周）缓解，没有静脉放血情况下HCT<45％，PLT≤400×10^9^/L，WBC<10×10^9^/L；
	③无疾病进展，没有任何出血或血栓事件；
	④骨髓组织学缓解，按年龄校正后的骨髓增生程度正常，三系高度增生消失，无>1级的网状纤维（欧洲分级标准）
部分缓解（PR）	以下4条必须全部符合：
	①包括可触及的肝脾肿大等疾病相关体征持续（≥12周）消失，症状显著改善（MPN-SAF TSS积分下降≥10分）；
	②外周血细胞计数持续（≥12周）缓解，没有静脉放血情况下HCT<45％，PLT≤400×10^9^/L，WBC<10×10^9^/L；
	③无疾病进展和任何出血或血栓事件；
	④没有骨髓组织学缓解，存在三系高度增生
无效（NR）	疗效没有达到PR
疾病进展（PD）	演进为真性红细胞增多症后骨髓纤维化（post-PV MF）、骨髓增生异常综合征或急性白血病

注：MPN-SAF TSS：骨髓增殖性肿瘤总症状评估量表；HCT：红细胞压积
